# Management of severe acute respiratory distress syndrome: a primer

**DOI:** 10.1186/s13054-023-04572-w

**Published:** 2023-07-18

**Authors:** John C. Grotberg, Daniel Reynolds, Bryan D. Kraft

**Affiliations:** Division of Pulmonary and Critical Care Medicine, Washington University School of Medicine, 660 S. Euclid Ave, St. Louis, MO 63110 USA

**Keywords:** Acute respiratory distress syndrome, Extracorporeal membrane oxygenation, Positive end expiratory pressure, Driving pressure, Mechanical power, Electrical impedance tomography, Esophageal manometry, Acute cor pulmonale

## Abstract

This narrative review explores the physiology and evidence-based management of patients with severe acute respiratory distress syndrome (ARDS) and refractory hypoxemia, with a focus on mechanical ventilation, adjunctive therapies, and veno-venous extracorporeal membrane oxygenation (V-V ECMO). Severe ARDS cases increased dramatically worldwide during the Covid-19 pandemic and carry a high mortality. The mainstay of treatment to improve survival and ventilator-free days is proning, conservative fluid management, and lung protective ventilation. Ventilator settings should be individualized when possible to improve patient-ventilator synchrony and reduce ventilator-induced lung injury (VILI). Positive end-expiratory pressure can be individualized by titrating to best respiratory system compliance, or by using advanced methods, such as electrical impedance tomography or esophageal manometry. Adjustments to mitigate high driving pressure and mechanical power, two possible drivers of VILI, may be further beneficial. In patients with refractory hypoxemia, salvage modes of ventilation such as high frequency oscillatory ventilation and airway pressure release ventilation are additional options that may be appropriate in select patients. Adjunctive therapies also may be applied judiciously, such as recruitment maneuvers, inhaled pulmonary vasodilators, neuromuscular blockers, or glucocorticoids, and may improve oxygenation, but do not clearly reduce mortality. In select, refractory cases, the addition of V-V ECMO improves gas exchange and modestly improves survival by allowing for lung rest. In addition to VILI, patients with severe ARDS are at risk for complications including acute cor pulmonale, physical debility, and neurocognitive deficits. Even among the most severe cases, ARDS is a heterogeneous disease, and future studies are needed to identify ARDS subgroups to individualize therapies and advance care.

## Introduction

The acute respiratory distress syndrome (ARDS), first described in 1967 [1], is a common cause of respiratory failure in the ICU. There are approximately 190,000 ARDS cases annually in the USA alone, although cases skyrocketed in 2020 due to the COVID-19 pandemic [2, 3]. ARDS pathophysiology is rooted in the disruption of the alveolar capillary barrier by inflammatory and oxidative insults. This results in the characteristic clinical (acute onset), radiographic (bilateral alveolar opacities), physiologic (reduced compliance, high shunt fraction), and histologic (classically diffuse alveolar damage) derangements. Severe ARDS, defined by an arterial partial pressure of oxygen (P_a_O_2_) to fraction of inspired oxygen (F_i_O_2_) ratio (P/F) ≤ 100, carries mortality close to 50% [2]. In moderate-to-severe ARDS, positive end expiratory pressure (PEEP) may confound the P/F ratio, and is addressed using the “P/FP ratio” ((P_a_O_2_*10)/(F_i_O_2_*PEEP)), with P/FP ≤ 100 defining severe ARDS [4]. The noninvasive ratio of pulse oximetric saturation (SpO_2_) to F_i_O_2_, or the “S/F ratio”, also correlates well to P/F ratios and is readily available at the bedside. Though not clearly defined, S/F ratios of < 89 to < 120 approximate severe ARDS [5–7].

**Fig. 1:**
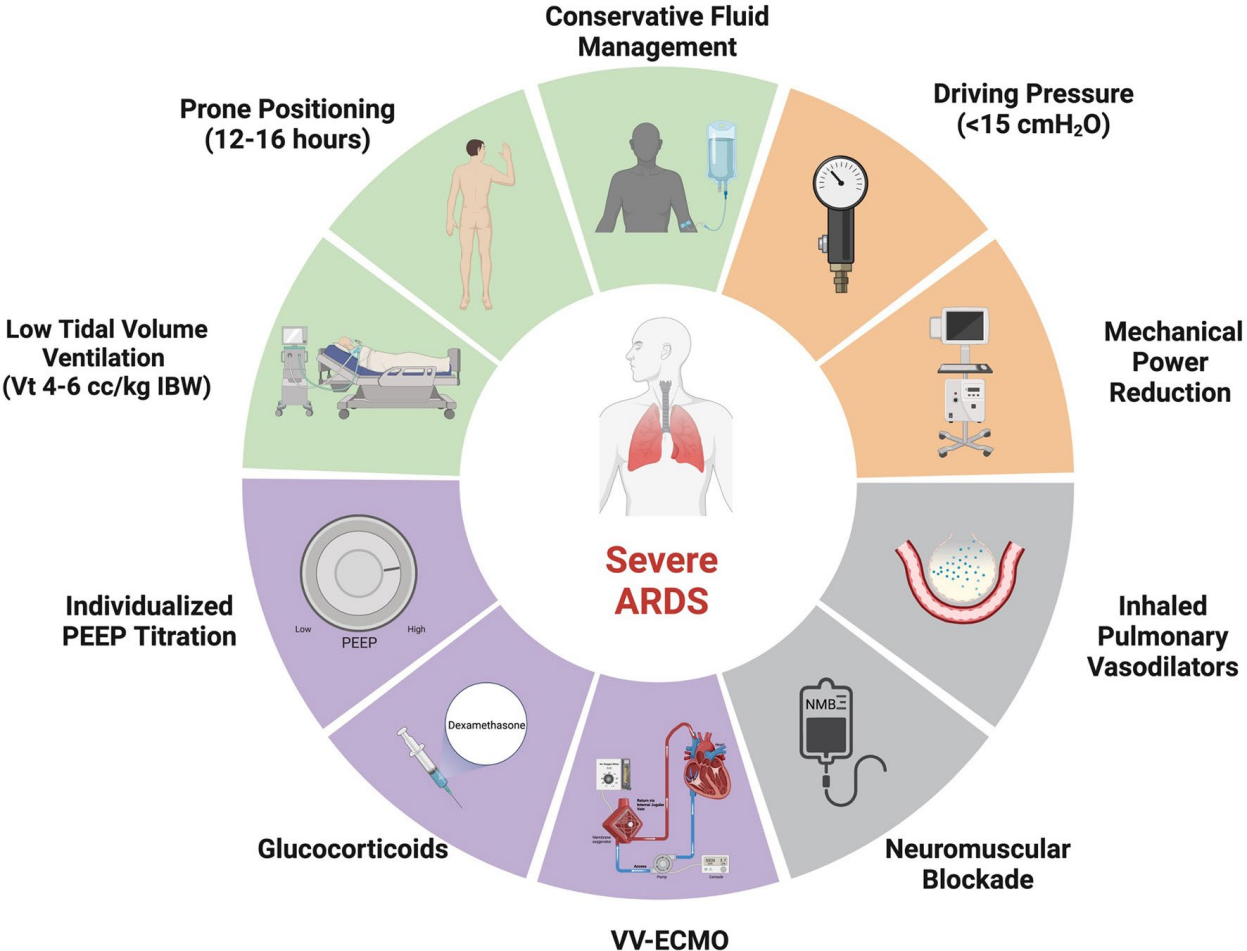
Severe ARDS Treatments. A schematic illustrating management strategies for severe ARDS and refractory hypoxemia. Green sections represent treatments that improve outcomes supported by prospective randomized controlled trials, the orange section represents a treatment that may improve outcomes based on retrospective data, the gray sections represent treatments that may improve oxygenation but have not demonstrated sustained clinical benefit in trials, and the purple sections represent treatments that likely derive benefit in a subset of patients. *ARDS* acute respiratory distress syndrome, *IBW* ideal body weight, *V*_t_, tidal volume; and V-V ECMO, veno-venous extracorporeal membrane oxygenation. Adapted from “Risk Factors of Dementia,” by BioRender.com (2023). Retrieved from https://app.biorender.com/biorender-templates

Patients with severe ARDS are at high risk for ventilator-induced lung injury (VILI) and may develop refractory hypoxemia and hypercapnia. Traditional treatment of severe ARDS is supportive, anchored by lung protective mechanical ventilation, proning, and conservative fluid management [8–10]. Adjunctive therapies (e.g., inhaled pulmonary vasodilators, glucocorticoids) can be used, and in select cases, patients may require veno-venous extracorporeal membrane oxygenation (V-V ECMO). This review will summarize the evidence-based management (Fig. 1) of severe ARDS emphasizing interventions that improve outcomes.

## Low tidal volume ventilation

Low tidal volume ventilation using either pressure-assist control (PC) or volume-assist control (VC) modes significantly improves mortality in ARDS [8, 11–13]. Neither mode is superior [14]. A VC mode controls tidal volume at the expense of controlling airway pressures, whereas a PC mode controls airway pressures at the expense of tidal volume and minute ventilation [15, 16]. Pressure regulated volume control (PRVC) is an adaptive mode that adjusts tidal volume for set pressure limits but may be insufficient in patients with high ventilatory drives [17].

The landmark ARMA trial demonstrated that a tidal volume of 6 cc/kg ideal body weight (IBW) compared to 12 cc/kg IBW reduced mortality (31% vs. 40%) and increased ventilator-free days [8]. While tidal volume ranged from 4 to 8 cc/kg in the trial, the goal tidal volume in the protocol was 4–6 cc/kg depending on plateau pressure (*P*_plat_). Average tidal volume in the intervention arm was 6.2 cc/kg over the first 5 days of trial enrollment and 6.5 cc/kg was used as a cut-off to designate study-site adherence. Physiologically, lower tidal volume ventilation reduces driving pressure, mechanical power, and the risk of volutrauma on the ARDS lung [18–20]. However, low tidal volumes (4–6 cc/kg) may still result in barotrauma, particularly in poorly compliant lungs. Barotrauma might be mitigated by further reducing tidal volumes (to lower airway pressures) in a VC mode, or with a PC mode. While low tidal volume ventilation improves mortality in ARDS, it may be poorly tolerated in some patients, leading to increased ventilator asynchrony and deeper sedation.

## Ventilator asynchrony

Patient-triggered modes of mechanical ventilation reduce work of breathing assuming matching between patient respiratory efforts and ventilator-delivered breaths [21]. Asynchrony events are common and may worsen outcomes if frequent. Asynchrony events can be quantified by the asynchrony index (AI), defined as the number of asynchrony events divided by the sum of the number of ventilatory cycles. In one study, 24% of the patients had an AI > 10% [21]. Evidence suggests AI > 10% may be associated with increased ICU and hospital mortality [22].

Common asynchronies include triggering asynchrony, cycling asynchrony and flow asynchrony. Ineffective triggering occurs when patient respiratory efforts do not result in ventilator-delivered breaths and is improved by increasing the trigger sensitivity of the ventilator or by using a flow-triggered. When ineffective triggering is due to excess intrinsic PEEP, efforts are directed to reduce intrinsic PEEP, or increase external PEEP to ~ 75% of the intrinsic PEEP to decrease the pressure gradient required by the patient to trigger the ventilator [23, 24]. Reverse triggering is seen in deeply sedated patients in which mechanical insufflation triggers a muscular effort, generating a “patient-triggered” breath and can be resolved by decreasing the level of sedation or by adding a neuromuscular blocking agent [24]. Cycling asynchronies occur cycling from the inspiratory to expiratory phase and may be premature or delayed. In premature cycling, a patient’s respiratory effort continues during the expiratory phase resulting in double-triggering and breath stacking. This is addressed by increasing the inspiratory time in PC or by increasing the tidal volume or decreasing the flow in VC. The opposite occurs during delayed cycling and is remedied by decreasing the inspiratory time in PC or increasing the flow in VC. Finally, flow asynchronies occur when patient flow demand does not match that of the ventilator. Flow starvation more often occurs in VC where patients exhibit excessive ventilatory demand and typically “suck down” the pressure–time wave form. Increasing the flow or changing to a PC mode can improve asynchrony. Conversely, excessive flow can be improved by decreasing the flow in VC or decreasing the inspiratory pressure in PC [23].

## Positive end expiratory pressure

PEEP opens collapsed alveoli allowing for recruited lung to participate in gas exchange and reduces alveolar overdistension by increasing distribution of the tidal breath. There is no clear mortality benefit in ARDS when comparing high PEEP to low PEEP strategies in all patients receiving low tidal volume ventilation, however, there may be a benefit in patients with moderate-to-severe ARDS, particularly patients who are PEEP-responsive (defined as an increase in P/F > 25 mm Hg after higher PEEP) [25–33]. Because of significant heterogeneity in ARDS, different phenotypes may respond differently to PEEP [34], therefore, clinicians should monitor oxygenation and lung compliance during titration. PEEP titration is performed by making stepwise increases in PEEP followed by small decremental changes of 2 cm H_2_O every 2–5 min while checking *P*_plat_ and monitoring changes in lung compliance. If a patient’s oxygenation or lung compliance worsen with increased PEEP, the PEEP is too high, whereas if they improve, the titration can continue until no further improvement is observed.

**Fig. 2:**
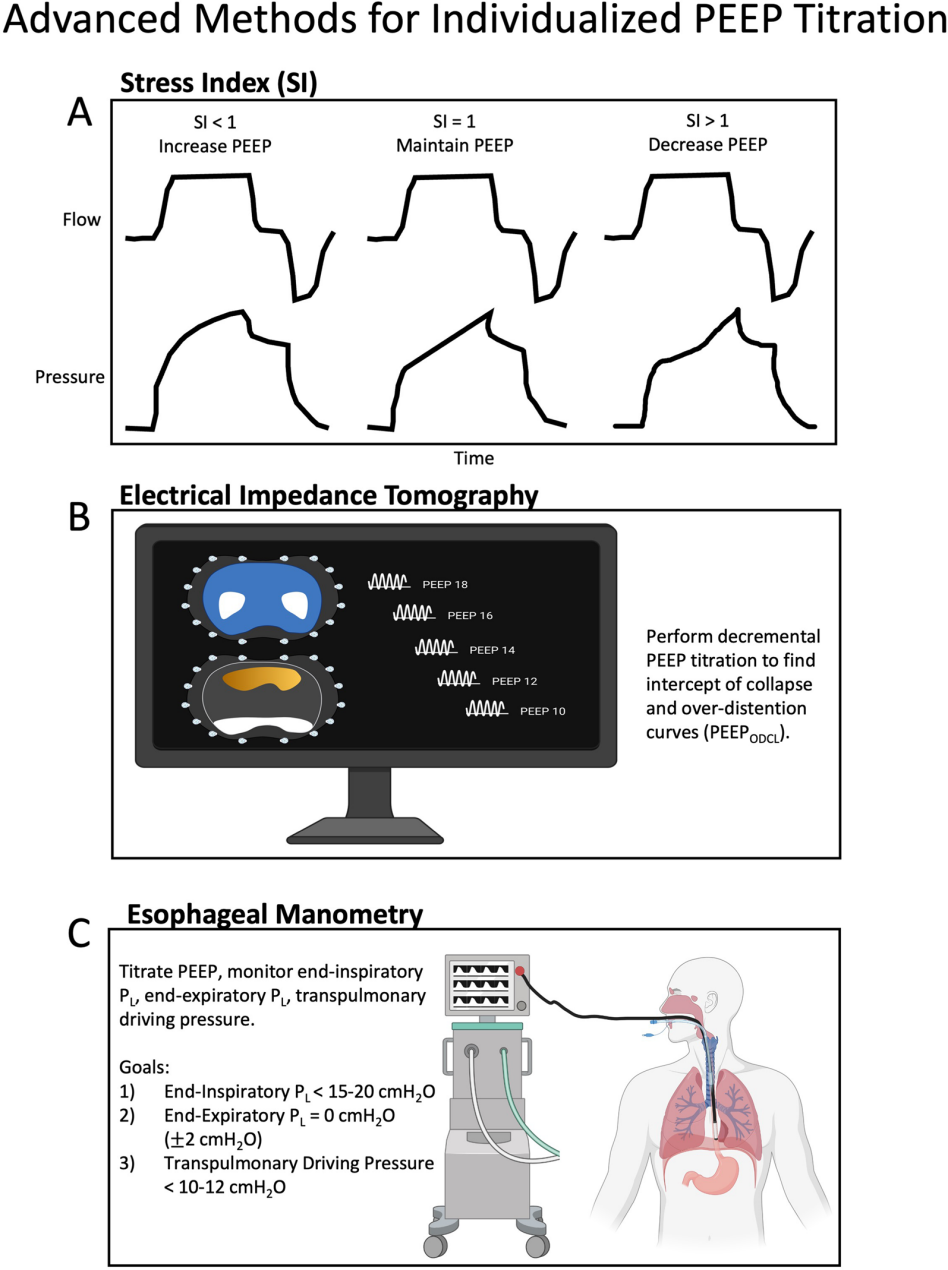
Advanced methods of PEEP titration. **A** The stress index, based on the pressure–time curve during constant flow (square-wave) volume-control ventilation. **B** Electrical impedance tomography with a proposed decremental PEEP titration. The top image depicts global tidal impedance where white indicates the highest volume change, and the bottom image depicts areas of alveolar over-distension (orange) and collapse (white). **C** Esophageal manometry and associated transpulmonary pressure targets. PEEP_ODCL,_ PEEP with least over-distended and collapsed lung; *P*_L_, transpulmonary pressure; SI, stress index. Created with BioRender.com

More advanced methods for individualizing PEEP include the stress index (SI), electrical impedance tomography (EIT), and esophageal pressure (*P*_es_) guidance (Fig. 2). The SI is based on the pressure–time curve during constant flow (square-wave) volume-control ventilation. A linear pressure rise suggests recruited alveoli without overdistension (SI = 1). Increasing compliance as the lungs are inflated (concave down waveform, SI < 1) suggests tidal recruitment, and benefit from increased PEEP. Conversely, decreasing compliance as the lungs are inflated (concave upward waveform, SI > 1) suggests overdistension, and benefit from decreased PEEP (Fig. 2a) [35]. SI is not superior to other PEEP titration methods [36, 37]. EIT determines the PEEP with the least overdistended and collapsed lung (PEEP_ODCL_) (Fig. 2b) [38–41]. In a study of severe ARDS, EIT-guided PEEP titration improved oxygenation, compliance and driving pressure [38]. Finally, esophageal manometry can be used to guide PEEP and operates under the assumption that the esophageal pressure (*P*_es_) is equivalent to the intrapleural pressure (*P*_pl_). PEEP is titrated to a transpulmonary pressure (*P*_L_) of 0 cm H_2_O, where *P*_L_ = *P*_ao_ − *P*_es_, and *P*_ao_ is the airway pressure [42]. *P*_Plat_ and PEEP can represent *P*_ao_ as the alveolar distending pressure at end-inspiration or end-expiration, respectively. In the EPVent trial, *P*_es_-guided PEEP titration improved oxygenation, however, when compared to empiric high PEEP in the EPVent-2 trial, there was no difference in clinical outcomes [43, 44]. A post hoc analysis of the EPVent-2 trial demonstrated that PEEP titrated to an end-expiratory *P*_L_ of 0 cm H_2_O was associated with greater survival than more positive or more negative values [45]. Ideal goals of esophageal manometry to guide PEEP include (1) end-inspiratory *P*_L_ < 15–20 cm H_2_O, (2) end-expiratory *P*_L_ = 0 cm H_2_O (± 2 cm H_2_O), and (3) transpulmonary driving pressure (end-inspiratory *P*_L_—end-expiratory *P*_L_) < 10–12 cm H_2_O (Fig. 2c) [42]. A newer and elegant method of determining lung recruitability by PEEP is the recruitment-to-inflation ratio, where a ratio ≥ 0.5 suggests lung recruitability at higher PEEP [46].

Regardless of the method used for PEEP titration and the metric(s) used assessing efficacy, monitoring hemodynamic responses is also necessary. PEEP can decrease cardiac output (by decreasing preload and increasing RV afterload), which can decrease DO_2_ despite an increase in oxygen saturation. Conversely, PEEP can reduce LV afterload [47]. Therefore, individualized PEEP titrations should consider oxygenation and driving pressure as well as hemodynamics.

## Recruitment maneuvers

A recruitment maneuver is a technique to increase the airway pressure in the lungs temporarily. Common methods used include sustained inflation (e.g. 35–40 cm H_2_O for 30–40 s in CPAP mode with a RR of 0) or a stepwise increase in PEEP followed by a decremental PEEP titration [35]. The physiologic rationale of a recruitment maneuver is to provide static or dynamic inflation at very high pressures for a short period of time to recruit alveolar units to participate in gas exchange and improve respiratory system compliance. Most lung recruitment occurs in the first 10 s of sustained inflation, while hemodynamic instability occurs after 10 s [48]. The effects of increasing PEEP likely stabilize after 11–20 breaths [49]. Recruitment maneuvers have been shown to improve oxygenation, however, have not been shown to improve mortality, and may actually be injurious [50–54]. In one study, 22% of patients who received recruitment maneuvers developed non-sustained hypotension and/or hypoxemia [54, 55]. In the ART trial, patients received a 4-min recruitment maneuver in a stepwise fashion (PC with PEEP at 25 cm H_2_O for 1 min, 35 cm H_2_O for 1 min, and 45 cm H_2_O for 2 min) followed by decremental PEEP. The recruitment maneuver strategy was modified mid-enrollment due to 3 cardiac arrests observed in the experimental arm, and overall the experimental arm showed increased mortality [56]. There is significant heterogeneity amongst studies evaluating recruitment maneuvers, making meta-analyses challenging to interpret [51]. Though some patients may show improved oxygenation with a recruitment maneuver, evidence suggests that there is no mortality benefit, and there may be associated harm. While not recommended routinely, select patients may respond favorably. If used, a stepwise increase in PEEP followed by decremental PEEP titration may be more effective [57], though more modest levels of PEEP should be used (20–25 cm H_2_O). Sustained inflation should be avoided to reduce the risk of hemodynamic instability.

## Driving pressure

In contrast to adjusting tidal volume for IBW, driving pressure adjusts tidal volume for compliance, and is the change in tidal volume relative to the static compliance of the respiratory system (*V*_t_/*C*_RS_), or the pressure differential required to inflate the lungs (*P*_plat_–PEEP). High driving pressures (> 15–17 cm H_2_O) are independently linked to ARDS mortality [58–62]. Amato et al. re-analyzed data from 3562 patients from 9 trials and found driving pressure was the variable that best stratified risk; reductions in driving pressure were strongly associated with increased survival [58]. The association between driving pressure and mortality was also observed in the LUNG SAFE study [2]. Newer analyses suggest that the mortality benefit seen in lowering tidal volume varies with respiratory system compliance, with greater benefit seen in patients with higher lung elastance [61, 62]. Lowering tidal volume to reduce driving pressure resulted in the greatest benefit in patients with low lung compliance. Optimizing ventilator settings to achieve a driving pressure < 15 may be the preferred target [2, 58, 59, 63]. There are ongoing clinical trials to investigate a driving pressure-driven approach to ventilator management [18].

## Airway pressure release ventilation

Airway pressure release ventilation (APRV) is an alternative mode of mechanical ventilation used to treat refractory hypoxemia and ARDS. APRV is a pressure-limited mode that cycles between two levels of CPAP. A higher airway pressure (P-high) is set for a certain time (*T*-high) and a lower airway pressure (*P*-low) (often set at 0 cm H_2_O) is set for a shorter time (*T*-low). APRV utilizes an inverted inspiration:expiration ratio, as the majority of spontaneous breathing is accomplished during *T*-high, with the higher pressure *P*-high theoretically allowing for recruitment of collapsed alveoli, and *T*-low allowing for ventilation and complete exhalation [64–66]. The proposed benefits to APRV include allowing for spontaneous breathing, decreased work of breathing, and less dyssynchrony (and therefore less use of sedatives and paralytics). It is also thought that higher mean airway pressures may improve oxygenation when compared to more conventional modes of mechanical ventilation [66]. While APRV may increase mean airway pressures there is less control over tidal volume and minute ventilation. Some patients may also require deep sedation and/or paralysis, thereby eliminating spontaneous breathing, compromising adequate ventilation. These issues may be overcome using time-controlled adaptive ventilation (TCAV), where T-low is set to terminate at 75% of the expiratory flow peak, maintaining adequate alveolar inflation during the release phase. If a patient requires a higher minute ventilation, *T*-high is reduced to increase the frequency of releases while *T*-low remains set based on expiratory flow dynamics [67, 68]. Despite its use in ARDS, high quality evidence favoring APRV is lacking, and the available studies reported mixed results. A systematic review and meta-analysis of eight studies found that use of APRV in critically ill adults with acute hypoxemic respiratory failure was associated with improved mortality and oxygenation, although the studies were small, single-center studies [69]. Another systematic review and meta-analysis of six studies with 375 patients found that APRV was associated with improved oxygenation and decreased ICU length of stay, but had no effect on mortality [64]. More recently, a randomized controlled trial of 90 adult patients with COVID-19 related ARDS compared APRV to conventional low tidal volume ventilation and found that APRV was not associated with improvements in ventilator-free days or mortality [70]. Larger, multicenter, randomized studies are needed to further clarify if APRV is beneficial in patients with severe ARDS (or in ARDS subgroups) compared with conventional ventilation.

## High frequency oscillatory ventilation

High frequency oscillatory ventilation (HFOV) is a mode of IMV that employs a constant airway pressure with oscillations at extreme respiratory frequencies (e.g., 5–15 Hz or 300–900 breaths per minute), delivering tidal volumes well below that of anatomical dead space [71, 72]. Gas exchange is by convection and diffusion: In large airways, convection predominates, where gas flow is dependent on turbulent flow, bulk convection, and central airway oscillatory pressure. In the lung periphery and alveolar units, diffusion predominates, where gas flow is dependent on Taylor dispersion, collateral ventilation, Pendelluft, and cardiogenic mixing. Higher oscillatory pressures recruit atelectatic alveoli but are dampened in aerated alveoli. In the small airways and mid-lung zones, both convention and diffusion direct gas flow and are dependent on turbulence, peripheral airways resistance, Pendelluft, and asymmetric inspiratory and expiratory velocity profiles [72]. While HFOV was previously considered a rescue mode of ventilation for severe ARDS, its use has fallen out of favor. Previous studies found mixed results among patients with moderate-to-severe ARDS [73–75], and a larger trial of 548 patients with moderate-to-severe ARDS demonstrated higher in-hospital mortality in patients randomized to HFOV compared with conventional high PEEP/low tidal volume ventilation [76]. However, in a meta-analysis of four studies (1552 patients total) comparing HFOV to conventional IMV, the association of HFOV on 30-day mortality varied with severity of hypoxemia: For patients with severe ARDS, HFOV was associated with improved mortality, whereas in patients with mild-to-moderate ARDS (P/F > 100), HFOV was associated with worsened mortality [77]. Though societies recommend against routinely using HFOV in patients with moderate-to-severe ARDS [78], there may be select patients with severe ARDS who benefit.

## Mechanical power

Mechanical power is the mechanical energy delivered from the ventilator to the respiratory system and has been hypothesized as a unifying driver of VILI [20]. Patients with severe ARDS receive mechanical ventilation with higher mechanical power than mild or moderate ARDS, though it is unclear if this is correlative or causative of further lung injury [79]. The power equation tidal volume, elastance, inspiratory and expiratory time, airway resistance, PEEP, and respiratory rate. This mathematical representation, however, does not necessarily address how energy is distributed to the lung parenchyma versus the respiratory system as a whole [80, 81]. Other simplified versions of the mechanical power equation have been derived using parameters easily measured at the bedside. The most clinically useful equation is $\mathrm{MP}=0.098\times {V}_{\mathrm{t}}\times \mathrm{RR}\times \left({P}_{\mathrm{peak}}-\frac{1}{2}\mathrm{DP}\right)$, where MP is mechanical power, *V*_t_ is tidal volume, RR is respiratory rate, *P*_peak_ is peak pressure, and DP is the driving pressure. Using this representation, an analysis of two cohorts of 8207 patients with ARDS showed that higher mechanical power (> 17.0 J/min) was independently associated with higher ICU-, hospital- and 30-day mortality and decreased ventilator-free days, even in patients receiving low tidal volumes [82]. Using a simpler model, Costa et al. also showed that driving pressure and RR ($\left(4\times \mathrm{DP}\right)+\mathrm{RR}$) was equivalent to mechanical power and associated with mortality [83]. This suggests that driving pressure and RR may be the more important variables of VILI.

## Proning

Prone ventilation improves oxygenation and ventilatory mechanics in many patients with severe ARDS [84–87]. There is often significant heterogeneity of pulmonary edema, consolidation, and atelectasis affecting dorsal lung regions. Proning improves heterogeneity allowing for increased lung recruitment, ventilation-perfusion matching, and decreased overdistension and lung stress. These physiologic effects have been demonstrated in animal models using electrical impedance tomography (EIT) [88, 89]. The PROSEVA trial is the most notable study of early proning in patients with moderate-to-severe ARDS (P/F < 150, FIO_2_ ≥ 60%). 28-day mortality in the proning group was 16% compared to 32.8% in the supine group (p < 0.001), and 90-day mortality in the proning group was 23.6% compared to 41% (*p* < 0.001). The average duration per proning session was 17 h and each patient underwent 4 proning sessions on average [9]. Meta-analyses of proning trials have shown improved oxygenation and improved mortality when proning sessions last ≥ 12 h [90–92]. Proning is generally indicated in moderate-to-severe ARDS (P/F < 150) after appropriate ventilator optimization. While paralysis may help to facilitate proning safely, it is not required. In PROSEVA, patients continued proning sessions until supine oxygenation improved to a P/F ≥ 150 with a PEEP ≤ 10 cm H_2_O and an FiO_2_ ≤ 0.6; therefore, smaller improvements in patient oxygenation should not necessarily halt proning. If oxygenation does not improve, patients may still benefit from improved respiratory mechanics and reduced lung stress, as the mortality benefit was not directly linked to improved oxygenation [93]. This may suggest static compliance, rather than P/F, is the more physiologically relevant proning endpoint [94]. However, robust data are lacking to support compliance-guided proning strategies.

## Fluid management

Acute lung injury during ARDS may be exacerbated by fluid overload. A landmark trial conducted by the ARDS Network (FACTT) compared two fluid management strategies in ARDS: a “conservative” strategy and a “liberal” strategy [10]. Treatment protocols consisted of combinations of IV fluids, diuretics, or inotropes based on the CVP or PAOP, cardiac output, and the presence or absence of shock and oliguria. While there was no effect on mortality, patients treated with conservative fluid strategy (goal CVP < 4 mm Hg and PAOP < 8 mm Hg in the presence of effective circulation) had less fluid accumulation and increased ventilator-free and ICU-free days.

Non-invasive methods, namely point-of-care ultrasonography (POCUS), can also be used to monitor hemodynamics and intravascular volume status. Venous congestion may be demonstrated by inferior vena cava (IVC) dilation with poor respiratory variability and S-wave reversal in the hepatic veins while low static filling pressures may be seen with a small IVC and a small, hyperdynamic LV cavity [95, 96]. An *E*/*E*’ ratio > 15 is associated with increased left-sided filling pressures, while an *E*/*E*’ ratio < 8 is associated with normal left-sided filling pressures, particularly when coupled with lung ultrasonography [97, 98]. Stroke volume and cardiac output can be evaluated using the left ventricular outflow tract velocity time integral (LVOT VTI) and diameter [96, 99]. IVC respiratory variation is a poor predictor of volume-responsiveness in patients with severe ARDS as this method was validated in patients receiving > 8 cc/kg IBW tidal volumes. Respiratory variation of LVOT VTI presents a better indicator of predicting fluid responsiveness, where a difference in 15 to 20% is associated with fluid responsiveness [96, 100].

## Glucocorticoids

The administration of empiric steroids for severe ARDS has remained controversial and clinical trial results have varied significantly. One trial conducted found moderate-dose methylprednisolone significantly reduced duration of mechanical ventilation, length of ICU stay, and ICU mortality [101]. However, a larger study in 2006 by the ARDS Network showed no clinical benefit in patients treated with steroids within 7 days of ARDS onset, and increased mortality in patients treated 14 days after ARDS onset [102]. More recently, the DEXA-ARDS trial studied patients with moderate-to-severe ARDS and found that patients who received dexamethasone experienced more ventilator-free days and lower mortality [103]. Dexamethasone has also been shown to improve overall mortality in patients with hypoxemia due to moderate or severe COVID-19 pneumonia [104–106].

Different ARDS subphenotypes display differing responses to corticosteroid treatment. A latent class analysis of the ARMA and ALVEOLI trials revealed the existence of two distinct phenotypes: (1) hyperinflammatory and (2) hypoinflammatory [34]. The hyperinflammatory phenotype exhibits a higher overall mortality, and in a retrospective analysis of COVID-19 ARDS, had improved mortality with steroids, while the hypoinflammatory group had worse mortality with steroids [107]. While the empiric use of glucocorticoids remains controversial in all patients with severe ARDS, there are likely select ARDS subgroups that derive benefit.

## Neuromuscular blockade

Neuromuscular blockade (NMB) improves oxygenation via several mechanisms. Paralysis decreases oxygen consumption, eliminates ventilator dyssynchrony, and improves thoracopulmonary compliance [108]. The ACURASYS trial in 2010 demonstrated a mortality benefit with 48 h of NMB with cisatracurium in patients with moderate-to-severe ARDS (P/F < 150) [109]. The larger multicenter ROSE trial in 2019 found no significant mortality benefit using NMB in moderate-to-severe ARDS [110]. However, patients already receiving NMB at the time of enrollment were excluded and it is possible that a subset of patients still benefit from NMB when deemed beneficial by clinician judgment. Additionally, in contrast to ACURASYS, the ROSE control arm received less sedation than the NMB group, which has been previously associated with improved ICU outcomes [111]. While it is evident that NMB improves oxygenation, it is controversial whether it confers a mortality benefit.

Prolonged use of NMB increases the risk of neuromuscular weakness and muscle loss, pressure injuries, and deep vein thromboses, and requires deep sedation which can increase delirium and neurocognitive impairment and decrease ventilator-free days [112, 113]. When using NMB agents, train-of-four (TOF) monitoring may be used to titrate to the lowest effective dose [114]. Deep sedation is also required during NMB and may be titrated using bispectral index (BIS) to a goal of 40 to 60 [115].

## Inhaled pulmonary vasodilators

Several trials have investigated the role of inhaled pulmonary vasodilators in ARDS, notably iNO and inhaled prostaglandins. Inhaled pulmonary vasodilators improve oxygenation and P/F ratio in most patients by improving ventilation-perfusion matching and may be used in patients with refractory hypoxemia [116, 117]. However, they do not improve mortality [116–119].

## Veno-venous extracorporeal membrane oxygenation

**Fig. 3:**
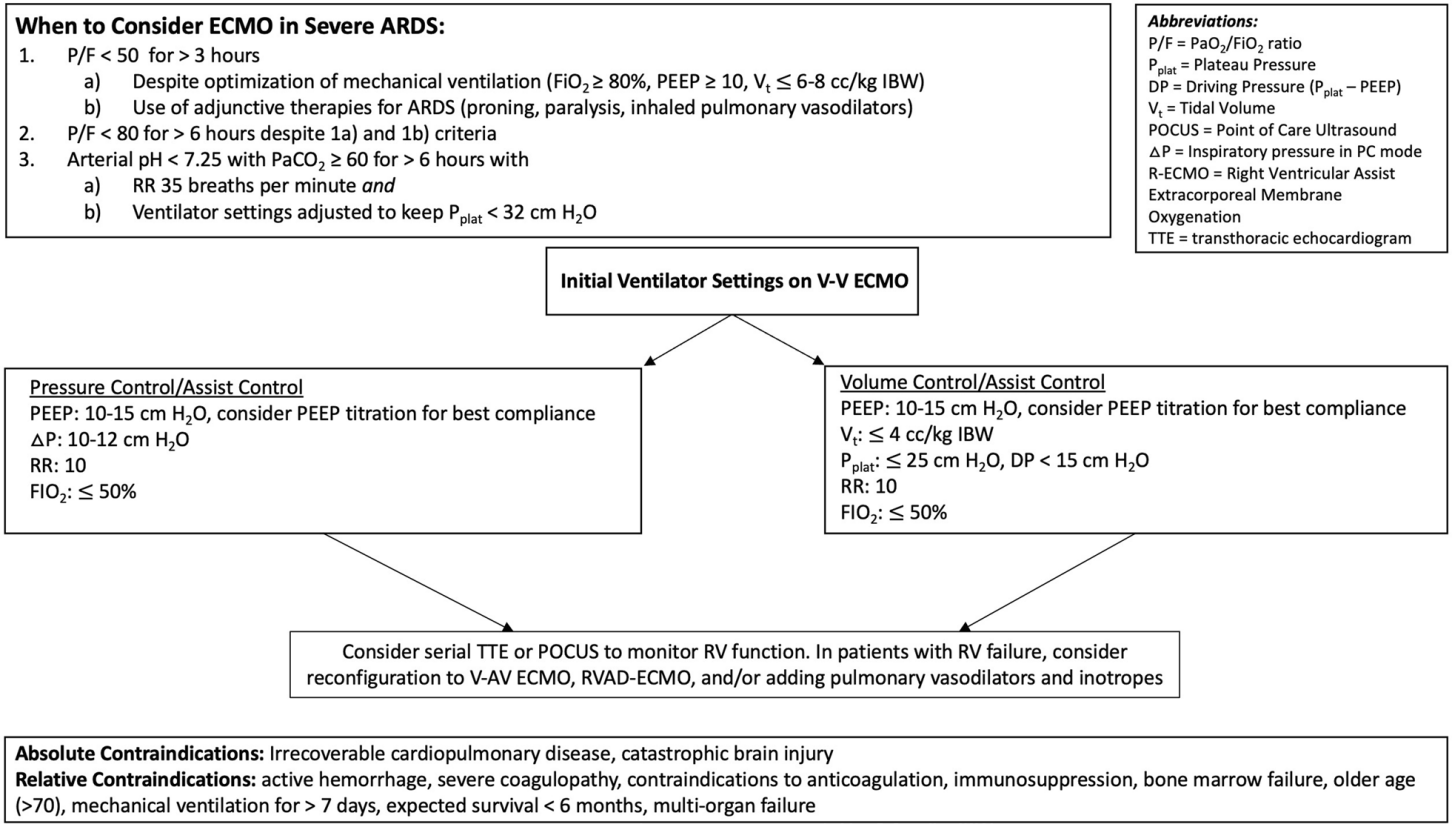
V-V ECMO considerations. A flowchart illustrating indications for veno-venous ECMO, initial ventilator management, monitoring of right ventricular function and contraindications to ECMO

V-V ECMO provides extracorporeal gas exchange in patients with refractory respiratory failure [120], and plays a critical role in the care of select patients with severe ARDS, though the selection criteria and timing of its use remain controversial. Studies have shown a wide array of outcomes when comparing ECMO to conventional management [121–123]. Two notable prospective randomized trials for V-V ECMO in ARDS were the CESAR trial and EOLIA trial. CESAR enrolled subjects with a Murray score ≥ 3 or pH < 7.2 despite optimal ventilator settings. CESAR randomized patients to transfer to an ECMO center, rather than ECMO itself. Of the patients that were transferred, 20% did not receive ECMO (instead they received optimized conventional mechanical ventilation), of which 82% survived. There was an overall survival benefit (63% versus 47%, *p* = 0.03) when transferred to an ECMO center [124]. EOLIA enrolled subjects with a P/F < 50 for > 3 h, P/F < 80 for > 6 h (with FIO_2_ > 80%) with optimal ventilator settings and adjunctive measures (paralysis, proning, inhaled pulmonary vasodilators), or pH < 7.25 and pCO_2_ > 60 while maintaining *P*_Plat_ < 32 and maximum RR 35 (Fig. 3). Though there was a non-significant trend toward improved mortality in the ECMO arm (*p* = 0.09), the study had an intention-to-treat design and 28% of the patients in the control group crossed over to receive salvage ECMO therapy, of which 43% survived [125]. The subgroup that benefitted most from ECMO were patients with excessive ventilatory pressures and refractory respiratory acidosis. A post-hoc Bayesian analysis and meta-analysis suggested ECMO may provide a ~ 10% mortality benefit [126, 127].

While optimal ventilator settings for patients on V-V ECMO are not clear, the use of ECMO allows for “lung rest” with dramatic reductions in driving pressure, *P*_Plat_, and mechanical power [128–132], which may reduce ongoing VILI [120, 124, 128, 131, 133, 134] (Fig. 3). Higher PEEP and lower driving pressure while on ECMO has been associated with improved mortality [135–137] and decreased cytokine release [138–141]. Optimal PEEP has been evaluated in small cohorts using EIT demonstrating that most patients require a PEEP of 10–15 cm H_2_O to minimize overdistension and atelectasis and improve compliance [142–144]. PEEP can also be titrated at the bedside to achieve optimal compliance.

## Acute cor pulmonale

**Fig. 4:**
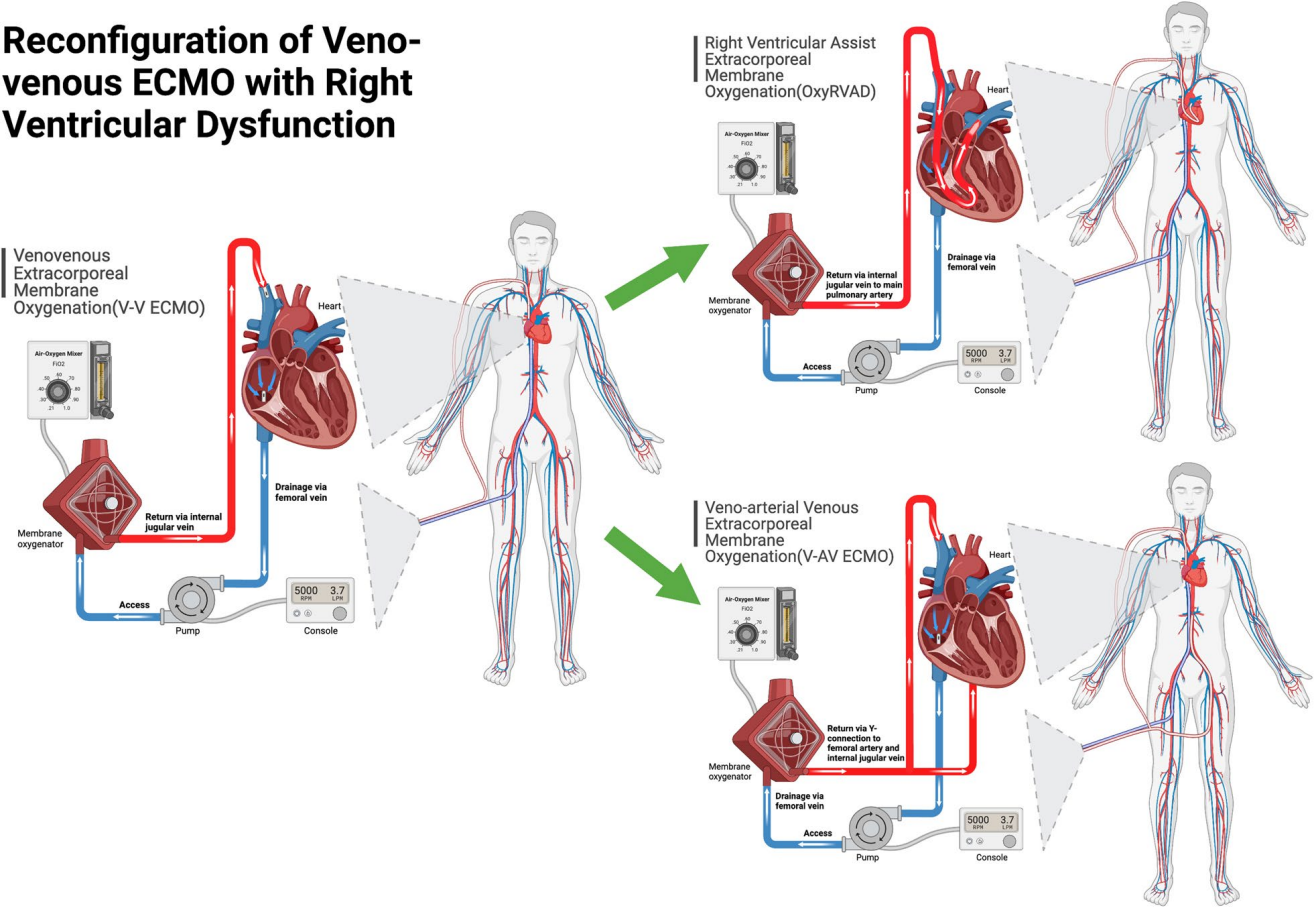
V-V ECMO configurations. A schematic illustrating the reconfiguration of conventional V-V ECMO to either right ventricular assist ECMO (OxyRVAD) or veno-arterial venous ECMO (V-AV ECMO). Adapted from “Extracorporeal Membrane Oxygenation (ECMO),” by BioRender.com (2023). Retrieved from https://app.biorender.com/biorender-templates

Acute cor pulmonale (ACP) is common in severe ARDS, with an estimated incidence of 25% [145], but may be higher in COVID-19 (~ 38%) [146]. The etiology of ACP is often multifactorial including pulmonary vascular dysfunction, regional hypoxemia with pulmonary vasoconstriction, and high mean airway pressures in the setting of poor lung compliance. Severe ACP, as defined by a right ventricular-to-left ventricular (RV/LV) ratio ≥ 1 with RV septal dyskinesia, is associated with even higher mortality [145]. Patients with severe ARDS should be serially monitored for the development of RV dysfunction via echocardiography or POCUS. If RV dysfunction develops, careful attention should be placed to intravascular volume status and cardiac output. Inhaled pulmonary vasodilators (e.g., iNO, epoprostenol, or systemic vasodilators (e.g., sildenafil), may be utilized to reduce pulmonary pressures. Inotropic agents may be used to augment cardiac output. The effects of PEEP on the pulmonary vascular resistance (PVR) and RV function may vary. The PVR-to-lung volume curve is generally U-shaped, with the lowest PVR at functional residual capacity [147]. Higher PEEP may induce more West zone 1 and 2 physiology resulting in increased PVR and RV dysfunction. However, hypoxic vasoconstriction in the pulmonary circulation also increases PVR, which may be addressed with higher PEEP [47, 148]. The clinician should carefully titrate PEEP understanding this nuance. Patients requiring V-V ECMO who develop ACP may be considered for circuit adjustment such as RV assist ECMO (OxyRVAD), where a return cannula is placed in the main pulmonary artery under transesophageal guidance to bypass the failing RV [149, 150] or veno-arterial venous ECMO (Fig. 4).

## ARDS survivorship

Survivors of severe ARDS are at increased risk for physical and neurocognitive sequelae that may persist for years. Common complications include vocal cord dysfunction and tracheal stenosis due to endotracheal tube pressure-related trauma, skin pressure injuries, frailty, neuromyopathies, and cognitive dysfunction [113]. One study of 109 ARDS survivors found persistent functional disability at one year after hospital discharge including abnormal pulmonary function testing, reduced 6-min walk distance, and reduced health-related quality of life. Moreover, ARDS severity predicted exercise capacity at 6 months [151]. Lower health-related quality of life was also seen in ECMO survivors [152]. Muscular weakness is common and affects long-term functioning. Acute skeletal muscle wasting occurs within one week, and is more pronounced in patients with multiorgan failure [153]. Patients who received corticosteroids and/or NMB are at higher risk for critical illness myopathy [113], and physical decline has been shown to persist at 5 years after discharge [154].

Neurocognitive dysfunction is also common after ARDS and data suggests and > 50% of survivors have persistent cognitive impairment at one year [155, 156]. Psychiatric morbidities, including depression, post-traumatic stress disorder (PTSD), anxiety and suicidality also occur at higher frequencies after ARDS [113].

## Conclusion

Severe ARDS carries a high morbidity and mortality, and refractory hypoxemia can prove challenging to manage. Low tidal volume ventilation, proning, conservative fluid management, and individualized PEEP titration to minimize driving pressure improve outcomes and are the mainstays of severe ARDS therapy. Optimizing ventilator-lung mechanics as they relate to mechanical power and driving pressure may further induce secondary VILI. Patients with refractory hypoxemia may benefit from inhaled pulmonary vasodilators and neuromuscular blockade, although these interventions have not been consistently shown to improve mortality. V-V ECMO likely confers a small (~ 10%) mortality benefit in a select subset of patients and can be considered on a case-by-case basis.

## Data Availability

Not applicable.

## Abbreviations

ACP: Acute cor pulmonale
AI: Asynchrony index
APRV: Airway pressure release ventilation
ARDS: Acute respiratory distress syndrome
C_RS_: Compliance of the respiratory system
CVP: Central venous pressure
DO_2_: Delivery of oxygen
DP: Driving pressure
EIT: Electrical impedance tomography
F_i_O_2_: Fraction of inspired oxygen
HFOV: High frequency oscillatory ventilation
IBW: Ideal body weight
ICU: Intensive care unit
iNO: Inhaled nitric oxide
IVC: Inferior vena cava
LV: Left ventricle
LVOT VTI: Left ventricular outflow tract velocity time integral
MP: Mechanical power
NMB: Neuromuscular blockade
OxyRVAD: Right ventricular assist extracorporeal membrane oxygenation
*P*_ao_: Airway pressure
P_a_O_2_: Partial pressure of oxygen in arterial blood
PAOP: Pulmonary artery occlusion pressure
PEEP: Positive end expiratory pressure
PEEP_ODCL_: PEEP with least overdistended and collapsed lung
*P*_es_: Esophageal pressure
*P*-high: The set high pressure during airway pressure release ventilation
*P*_L_: Transpulmonary pressure
*P*-low: The set low pressure during airway pressure release ventilation
POCUS: Point-of-care ultrasonography
*P*_peak_: Peak pressure
*P*_Pl_: Pleural pressure
*P*_plat_: Plateau pressure
PTSD: Post-traumatic stress disorder
PVR: Pulmonary vascular resistance
RR: Respiratory rate
RV: Right ventricle
SI: Stress index
*T*-low: The set time during the low pressure of airway pressure release ventilation
*T*-high: The set time during the high pressure of airway pressure release ventilation
V-AV ECMO: Veno-venoarterial extracorporeal membrane oxygenation
VILI: Ventilator-induced lung injury
*V*_t_: Tidal volume
V-V ECMO: Veno-venous extracorporeal membrane oxygenation
